# Chokeberry Extract and Its Active Polyphenols Suppress Adipogenesis in 3T3-L1 Adipocytes and Modulates Fat Accumulation and Insulin Resistance in Diet-Induced Obese Mice

**DOI:** 10.3390/nu10111734

**Published:** 2018-11-12

**Authors:** Na-Hyun Kim, Jonghwan Jegal, Yun Na Kim, Jeong-Doo Heo, Jung-Rae Rho, Min Hye Yang, Eun Ju Jeong

**Affiliations:** 1 Gyeongnam Department of Environment & Toxicology, Korea Institute of Toxicology, 17 Jegok-gil, Munsan-eup 52834, Korea; nhkim@kitox.re.kr (N.-H.K.); jdheo@kitox.re.kr (J.-D.H.); 2 College of Pharmacy, Pusan National University, Busan 46241, Korea; jhjegal@pusan.ac.kr; 3 Department of Agronomy and Medicinal Plant Resources, Gyeongnam National University of Science and Technology, Jinju 52725, Korea; skdbssk@hanmail.net; 4 Department of Oceanography, Kunsan National University, Kunsan 54150, Korea; jrrho@kunsan.ac.kr

**Keywords:** *Aronia melanocarpa*, polyphenols, anti-adipogenesis, adipogenic transcription factor, diet-induced obesity, insulin resistance

## Abstract

Berries of *Aronia melanocarpa* (chokeberry) are known to be a rich source of biologically active polyphenols. In the present study, the effects of seven anti-adipogenic polyphenolic phytochemicals isolated from *A. melanocarpa* methanol extract on adipogenic transcription factors were investigated. Amygdalin and prunasin were found to inhibit 3T3-L1 adipocyte differentiation by suppressing the expressions of PPARγ (peroxisome proliferator-activated receptor γ), C/EBPα (CCAAT/enhancer binding protein α), SREBP1c (sterol regulatory element binding protein 1c), FAS (fatty acid synthase), and aP2 (adipocyte fatty-acid–binding protein). *A. melanocarpa* extract-treated (100 or 200 mg/kg/day on body weight) high fat diet (HFD)-induced obese mice showed significant decreases in body weight, serum triglyceride (TG), and low-density lipoprotein cholesterol (LDLC) levels and improved insulin sensitivity as compared with HFD controls. This research shows *A. melanocarpa* extract is potentially beneficial for the suppression of HFD-induced obesity.

## 1. Introduction

Obesity is a chronic, relapsing, and multifactorial disorder with wide-ranging causes [1,2], and in Asian men is defined as a body mass index (BMI) of ≥30 kg/m^2^ [3]. Obesity is also known to be associated with diverse comorbidities including cardiovascular diseases, dyslipidemia, and type 2 diabetes [4]. Today obesity has reached epidemic proportions, and thus, has become a major global public health problem. The condition is the result of the excessive accumulation of adipocytes, which store and regulate energy in the form of triglycerides or free fatty acids [5]. Adipocyte growth occurs due to hypertrophy (an increase in cell size) and hyperplasia (an increase in cell number) [5,6], and white adipose tissue acts as a major endocrine organ by secreting protein signals and factors called adipokines [7]. Although the precise mechanism has not been delineated, it is generally held that the pathogeneses of complications related to obesity involve abnormal adipokine production by adipocytes [8].

Polyphenols are commonly distributed in fruits and vegetables and include a huge variety of compounds [9]. Furthermore, polyphenols have been shown to possess various beneficial biological effects, especially antioxidant activity [9,10], and because oxidative stress is considered to underlie the pathogeneses of many diseases, plants that produce polyphenolics have attracted the interest of nutritional scientists [11]. Oxidative stress and obesity have been proposed to be linked, and oxidative stress has been reported in obese individuals that have lost weight [12]. Accordingly, studies have been conducted to identify the therapeutic effects of polyphenol-rich plants, such as, grapes [13], cranberry [14], and green tea [15], with respect to the prevention and treatment of obesity and its metabolic complications.

Black chokeberry, *Aronia melanocarpa* (Michx.) Elliot, is a deciduous shrub native to eastern North America belonging to the genus Rosaceae [16], and continues to be used by Native Americans as an herbal medicine for treatment of colds [17], whereas in Russia and Eastern Europe, *A. melanocarpa* is traditionally used to treat hypertension and atherosclerosis [18]. Furthermore, scientific studies have shown the plant has hepatoprotective [19], antidiabetic and hypolipidemic [20], and cardiovascular-protective [21] effects. Moreover, these health benefits of chokeberries have been associated with their high polyphenolic contents, as they are rich sources of anthocyanins and proanthocyanidins, which have strong antioxidant properties [22]. This study was performed to identify the phenolics responsible for the anti-obesity effects of *A. melanocarpa*. In addition, we investigated the effect of a methanol extract of *A. melanocarpa* on body weight and serum lipid levels in high fat diet (HFD)-induced obese mice.

## 2. Materials and Methods

### 2.1. General Experimental Procedures

UV spectra were obtained in MeOH using a Varian Cary 50 spectrometer and IR spectra on a JASCO FT/IR 4100 spectrometer. All NMR spectra were recorded on a Varian VNMRS spectrometer (500 and 125 MHz for 1H and 13C NMR, respectively) in CDCl_3_. Proton and carbon chemical shifts were referenced versus 7.26 and 77.0 ppm, respectively. Electrospray ionization mass spectroscopy/mass spectroscopy (ESIMS/MS) spectra were acquired in enhanced product ion mode using an AB SCIEX QTRAP 3200 unit at an ion source potential of 5500 V, a declustering potential of 60 V, and a collision energy of 35 eV. HPLC was performed using a Varian Prostar system equipped with a 355 refractive index detector and either a YMC-pack ODS-H80 (5 μm, 150 × 4.6 mm) or a YMC-pack Si (5 μm, 250 × 10.0 mm) column.

### 2.2. Plant Material

*A. melanocarpa* (Michx.) Elliot berries were obtained from the Samheung Agricultural Corporation (Geochang, Korea) and identified by Professor Yang, Min Hye (College of Pharmacy, Pusan National University). A voucher specimen (GNP-78) has been deposited in the Laboratory of Pharmacognosy, College of Life Sciences, Gyeongnam National University of Science and Technology.

### 2.3. Extraction and Isolation of Polyphenols from Aronia Berries

Fruits were freeze dried for 5 days and dried fruits (0.5 kg) were extracted with 100% MeOH for 48 h, which was then filtered and concentrated in vacuo. The extract was then added to 1 L of water and sequentially partitioned with hexane (1 L), CH_2_Cl_2_ (1 L), ethyl acetate (1 L), and BuOH (1 L). A portion of the in vacuo concentrated BuOH fraction (5 g) was subjected to reversed-phase flash chromatography; elution was performed using a step gradient from 50% MeOH (BR1) to 100% MeOH (BR6) in H_2_O. Fraction BR3 was separated by reverse-phase preparative HPLC (YMC H80, 150 × 20 mm, 30% MeOH in H_2_O containing 0.05% TFA, at a flow rate of 5 mL/min) to afford a mixture of compounds **1** and **2**. These two compounds were separated by HPLC using a Phenomenex C6-phenyl column (250 × 10 mm) eluted with 15% ACN in H_2_O (0.1% formic acid) at a flow rate of 2 mL/min. Similarly, using the same HPLC conditions used to separate fraction BR3, compounds **3**–**7** were isolated and purified from fraction BR4.

### 2.4. Evaluation of Anti-Adipogenic Activities in 3T3-L1 Cells

#### 2.4.1. Cell Culture

Mouse embryo fibroblast 3T3-L1 cells were obtained from the American Type Culture Collection (Manassas, VA, USA) and incubated in Dulbecco’s modified Eagle’s medium (DMEM) supplemented with 10% bovine calf serum (BCS) and 100 IU/mL penicillin and 100 mg/mL streptomycin until confluent. Two days later (designated day 0), preadipocytes were stimulated to differentiate by transferring them to differentiation medium (DM) with DMEM containing 10% fetal bovine serum (FBS), 0.5 mM 3-isobutyl-1-methylxanthine, 10 μg/mL insulin, 1 μM dexamethasone, and penicillin/streptomycin for 3 day (days 0–2). During days 3 and 4, cells were maintained in DM (DMEM containing 10% FBS, 10 μg/mL insulin, and penicillin/streptomycin), and then cultured for a further 4 days in DM. Cells were then maintained at 37 °C in a humidified 95% air/5% CO_2_ atmosphere.

The purities of all 7 compounds were verified to be >98% by HPLC. Test compounds were dissolved in dimethyl sulfoxide (DMSO) to a final concentration of 0.1% in media and added to the cell cultures mentioned above for the entire 8-day culture period.

#### 2.4.2. Oil Red O Staining

Lipid droplets in cells were stained with ORO. On day 8, culture dishes were washed three times with PBS and attached cells were fixed with 10% formalin for 1 h at room temperature, washed once with PBS, stained with filtered ORO solution (6 parts saturated 0.6% ORO in isopropyl alcohol and 4 parts water) for 15 min at room temperature, rewashed twice with water for 15 min. and then visualized. To quantify intracellular lipids, ORO stained lipid droplets were dissolved with 4% Nonidet P-40 in isopropyl alcohol for 5 min and absorbances were measured spectrophotometrically at 544 nm.

#### 2.4.3. Measurement of Cell Proliferation

MTT assay was used to assess cell viability, in which the tetrazolium dye 3-(4,5-dimethylthiazol-2-yl)-2,5-diphenyl tetrazolium bromide (MTT) is reduced by cellular enzymes to its insoluble formazan. 3T3-L1 adipocytes were seeded in 96-well plates at 5 × 103 cells/well and incubated for 24 h. Cells were then treated with vehicle or compound for 24 or 48 h. Inhibitory effects on cell proliferation were assessed by adding MTT (final concentration 0.5 mg/mL in media) directly added to cultures and incubating at 37 °C for 4 h. Supernatants were then removed by aspiration and 100 μL of DMSO was added to dissolve the formazan produced. Absorbances were measured at 540 nm by using a microplate reader. Results are expressed as cell viabilities expressed as percentages of those of control cultures.

#### 2.4.4. Real-Time RT-PCR

Total RNA was extracted from 3T3-L1 cells using the RNease Plus Kit (QIAGEN Korea Ltd., Seoul, Korea). cDNA was synthesized with 1 μg of total RNA using the QuantiTech Reverse Transcription Kit (QIAGEN Korea), after which it was mixed with QuantiFast SYBR Green PCR master mix (QIAGEN Korea) with specific primers in a total reaction volume of 20 μL. The PCR specific primers used with QIAGEN kits for SYBR Green-based real-time RT-PCR were C/EBPα (NM_007678), C/EBPβ (NM_009883), C/EBPδ (NM_007679), SREBP1 (NM_011480), stearoyl coenzyme A desaturase 1 (SCD-1; NM_009127), fatty acid synthase (FAS; NM_007988), acetyl-CoA carboxylase (ACC; NM_133360), and glyceraldehyde 3-phosphate dehydrogenase (GAPDH; NM_008084) (all were obtained from QIAGEN Korea). Amplification cycles were carried out at 95 °C for 20 s, 60 °C for 20 s, and 72 °C for 20 s, and the last cycle was followed by a final extension step at 72 °C for 5 min. Quantitative SYBR Green real-time RT-PCR was performed using an Applied Biosystems 7300 Real-Time PCR System (Life Technologies Corporation, Carlsbad, CA, USA) and results were analyzed by performing comparative *C*_t_ quantifications. GAPDH was amplified as the internal control. *C*_t_ values of GAPDH were subtracted from the *C*_t_ values of target genes (Δ*C*_t_), and the Δ*C*_t_ values of treated cells were compared with those of untreated cells.

### 2.5. Evaluation of Anti-Obesity Effects in HFD-Induced Obese Mice

#### 2.5.1. Sample Preparations

Fresh *A. melanocarpa* fruits were washed and juice was obtained by screw compression (Dongnam Co. Ltd., Yangsan, Korea). Mean soluble solid (24° Brix) level in aronia juice was 56.81 ± 3.05%. The compressed extract was freeze-dried and weighed, followed by dissolved in 0.5% carboxymethyl cellulose (CMC).

#### 2.5.2. Animals and Diets

Animal experiments were carried out according to the guidelines issued by the Gyeongnam Department of Environment & Toxicology, Korea Institute of Toxicology on the Care and Use of Laboratory Animals (Certification No. KIT-1603-0004). Male C57BL/6J mice (3 weeks old) were purchased from Central Lab. Animal Inc. (Seoul, Korea). Animals were acclimatized for two weeks under a 12 h:12 h light–dark cycle at 20 ± 2 °C and an RH of 50 ± 5% with ad lib. access to food and water. Mice were divided into five groups, as detailed below. Animals were fed a normal diet containing 10% kcal fat (3.85 kcal/g) or a high-fat diet containing 60% kcal fat (5.24 kcal/g) (Research Diets Inc., New Brunswick, NJ, Canada). Food consumption in the HFD-fed groups was calculated every 7 days (amount of food intake = weight of a feeder − weight of 24 h after the measured feeder). *A. melanocarpa* extract dissolved in 0.5% CMC was orally administered (at 100 or 200 mg/kg) once a day for eight weeks. High-fat diet mice received either 15 mg/kg of Orlistat (Zenical^®^, Roche Pharm Ltd., Reinach, Switzerland) or 0.5% CMC (10 mL/kg) orally as positive or negative controls. A normal diet group was also treated with only 0.5% CMC (10 mL/kg body weight). The 5 study groups were as follows.

Group I (ND): received oral 0.5%-CMC and fed a normal diet (normal control group).

Group II (VC): received oral 0.5%-CMC and fed the HFD, used as a disease group (Negative control group).

Group III (PC): received Orlistat (15 mg/kg, p.o.) and fed the HFD (positive control group)

Group IV (T1): received *A. melanocarpa* extract (100 mg/kg, p.o.) and fed the HFD.

Group V (T2): received *A. melanocarpa* extract (200 mg/kgm, p.o.) and fed the HFD.

#### 2.5.3. OGTT and IPITT Tests

Oral glucose tolerance test (OGTT) and intraperitoneal insulin tolerance test (IPITT) tests were performed a week before sacrifice as previously described [23]. Briefly, for the OGTT test, 2 g/kg of glucose was orally administered after a 6-h fast. For the IPITT test, 1 IU/kg insulin was injected intraperitoneally (i.p.) after a 4-h fast. Blood glucose levels were monitored using an Accu-Chek Active Glucometer (Roche) at 0, 15, 30, 60, and 120 min after glucose or insulin treatment.

#### 2.5.4. Body and Adipose Tissue Weights Food Consumption and Biochemical Analysis of Serum Samples

Body weights were measured in a nonfasted state weekly. The food consumption measured per cage once a week. Food consumption was calculated as Food consumption (g) = Full feeder − Empty feeder/day/number of mice per cage. After 8 weeks of treatment, mice were sacrificed by cervical dislocation and epididymal and perirenal adipose tissues were dissected and weighed. Blood samples were obtained from the abdominal aorta, left in a separation tube at room temperature for 30 min, and centrifuged at 3000 rpm for 15 min. Serum levels of triglyceride (TG), total cholesterol (TC), low-density lipoprotein cholesterol (LDLC), and high-density lipoprotein cholesterol (HDLC) were measured using a Hitachi 7180 Automatic Analyzer (Hitachi High Technologies, Seoul).

### 2.6. Statistical Analysis

Results are presented as means ± SDs. Body weight data were analyzed by two-way ANOVA, whereas in vitro assay and in vivo biochemical parameters were analyzed by one-way ANOVA. Statistical significance was accepted for *p*-values < 0.05.

## 3. Results and Discussion

### 3.1. Isolation of Polyphenols from A. melanocarpa

**Figure 1:**
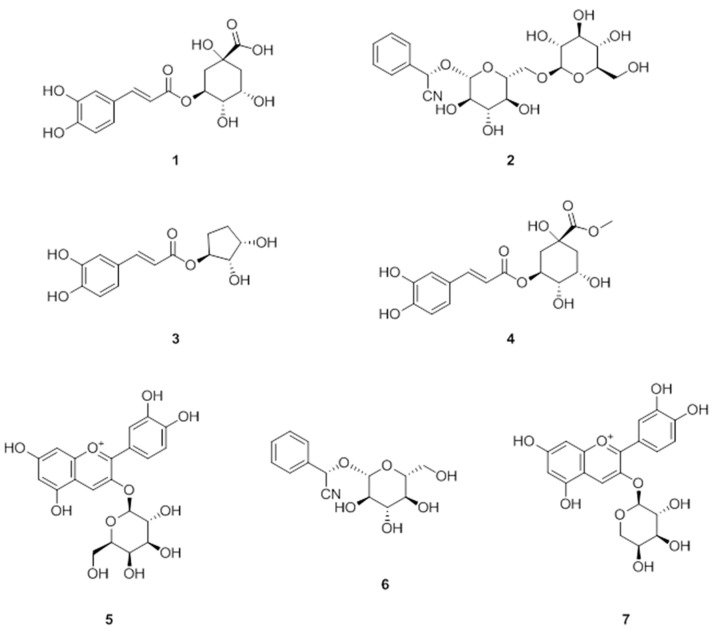
Chemical structure of compounds isolated from *Aronia melanocarpa* (Chokeberries). **1**: chlorogenic acid, **2**: amygdalin, **3**: 1-(3,4-dihydroxycinnamoyl cyclopenta-2,3-diol, **4**: methyl 3-*O*-caffeoylquinic acid, **5**: cyanidin-3-glucoside, **6**: prunasin, and **7**: cyanidin-3-xyloside.

*A. melanocarpa* is one rich in proanthocyanidins, anthocyanins, flavonols, and flavanones [17,24,25,26]. Its anthocyanins are mainly composed of a mixture of four cyanidin glycosides, that is, 3-galactoside, 3-glucoside, 3-arabinoside, and 3-xyloside [17]. Furthermore, previous studies have shown *A. melanocarpa* has beneficial effects on human health, with respect to the prevention of cardiovascular diseases, diabetes, cancer, and obesity. In this study, we evaluated the anti-obesity effects of an extract isolated from the fruits of *A. melanocarpa*, as described above. Fractions of the extract were also evaluated for their abilities to inhibit adipocyte differentiation. The *n*-BuOH fraction showed significant inhibitory activity (data not shown) and was further subjected to repeated column chromatography to yield seven compounds (Figure 1). The isolated compounds were identified as chlorogenic acid (**1**), amygdalin (**2**), 1-(3,4-dihydroxycinnamoyl cyclopenta-2,3-diol (**3**), methyl 3-*O*-caffeoylquinic acid (**4**), cyanidin-3-glucoside (**5**), prunasin (**6**), and cyanidin-3-xylosise (**7**), by comparing their spectroscopic data with those previously reported. An in vitro study was undertaken to investigate the effects of compounds **1**–**7** on adipogenic transcription factors in 3T3-L1 adipocytes.

### 3.2. Anti-Adipogenic Effects of the Seven Polyphenols on 3t3-L1 Preadipocytes

**Figure 2:**
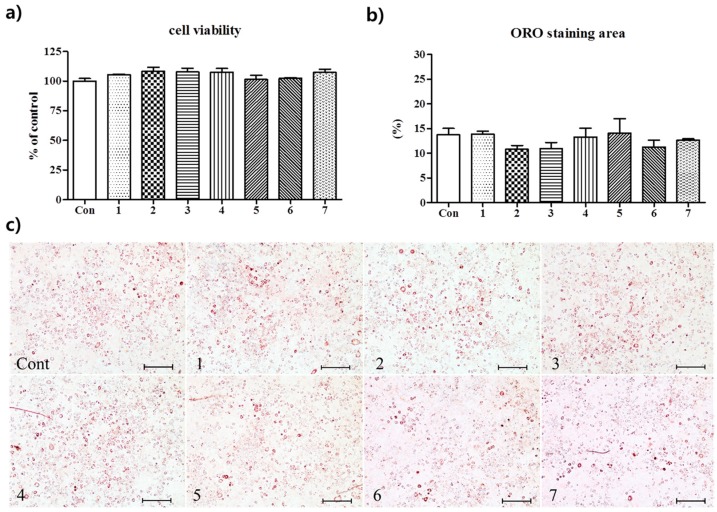
Suppression of the differentiation of 3T3-L1 preadipocytes by compounds **1**–**7** isolated from A. melanocarpa extract. (**a**) Cytotoxic effects compounds **1**–**7** on 3T3-L1 cells. Cells were exposed to the compounds at 10 μM. Results are the means ± SDs of triplicate experiments. (**b**) Anti-adipogenic effects of compounds **1**–**7** in 3T3-L1 cells. Cells were exposed to differentiation cocktail (0.5 mM IBMX, 10 μg/mL insulin, and 1 μM dexamethasone) in the presence or absence of samples at a concentration of 10 μM for eight days. Morphological changes were detected under a microscope and lipid accumulations were assessed using Nile Red fluorescent reagent. Results are the means ± SDs of triplicate experiments.

Confluent 3T3-L1 preadipocytes were treated with compounds **1**–**7** at the nontoxic concentration of 10 μM (no toxic effect was observed when 3T3-L1 preadipocyte were treated for 48 h; Figure 2a). The murine 3T3-L1 preadipocyte cell-line was chosen because differentiated 3T3-L1 cells have been commonly utilized as a cell model in adipose cell biology research [27]. In the present study, adipocyte differentiation was induced by adding 0.5 mM IBMX, 10 μg/mL insulin, plus 1 μM dexamethasone to culture medium. On culture day 8, lipid droplets were stained with Oil Red O (ORO), photographed under a phase-contrast microscope and quantified spectrophotometrically at 544 nm. As shown in Figure 2b,c, lipid contents were slightly diminished by treating 3T3-L1 cells with compounds **2**, **3**, or **6** without statistical significance.

**Figure 3:**
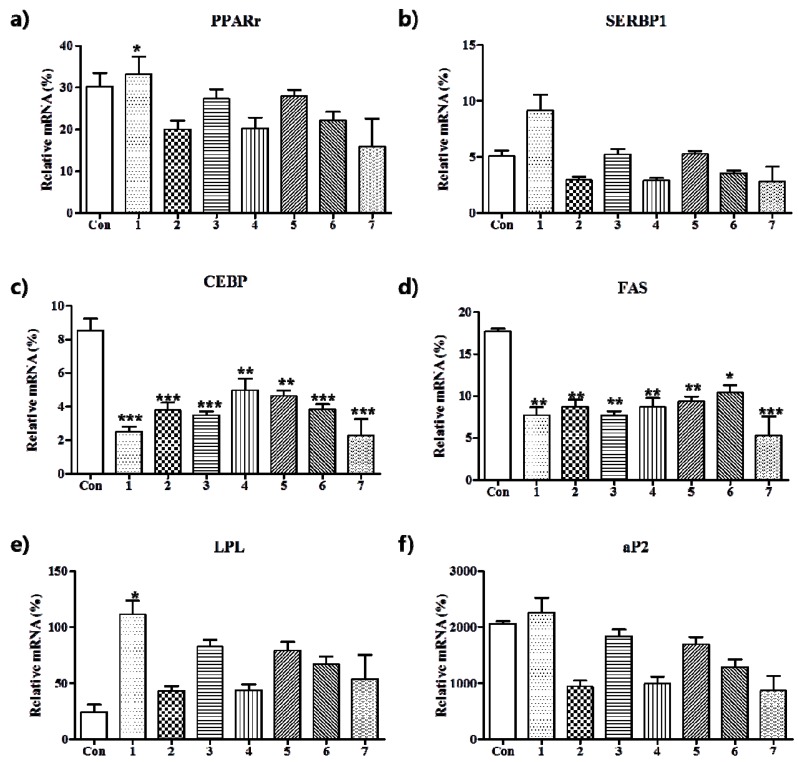
Inhibitions of the mRNA expressions of PPARγ (**a**), SREBP1 (**b**), C/EBPα (**c**), FAS (**d**), LPL (**e**), and aP2 (**f**) by compounds **1**–**7** in differentiated 3T3-L1 cells. Cells were exposed to differentiation cocktail (0.5 mM IBMX, 10 μg/mL insulin, and 1 μM dexamethasone) in the presence or absence compounds **1**–**7** at a concentration of 10 μM for eight days. Results are expressed as means ± SDs, *n* = 3 per group. * *p* < 0.05, ** *p* < 0.01, and *** *p* < 0.001 versus treatment naïve controls.

A series of programmed gene expressional changes in adipokines affect adipocyte differentiation [28]. Therefore, we investigated the anti-adipogenic effects of the seven compounds by determining the expressions of adipogenic transcription factors by quantitative real-time PCR. Adipocyte differentiation requires several transcription factors, including C/EBPα, PPARγ, and SREBP1c. According to the paradigm of adipocyte development, PPARγ initiate the differentiation cascade and C/EBP is important for maintaining the expression of PPARγ [29]. Transcription factors, such as SREBP1c and FAS, cannot promote adipogenesis in isolation, but when coexpressed on fibroblasts expressing PPARγ, adipocyte differentiation is enhanced [30]. In the present study, treatment of 3T3-L1 adipocytes with compounds **1**–**7** markedly lowered the expressions of C/EBP and FAS as compared with those observed in fully differentiated adipocytes (Figure 3). In addition, the gene expressions of PPARγ, SREBP1c, and aP2 in 3T3-L1 adipocytes were by **2**, **4**, **6**, and **7**, indicating the inhibition of adipocyte differentiation was mediated by the regulation of lipogenesis [31,32].

### 3.3. Anti-Obesity Effects of A. melanocarpa Extract in HFD-Induced Obese Mice

#### 3.3.1. Effects of *A. melanocarpa* Extract on Body Weight Gains and Adipose Tissue Weights in HFD-Induced Obese Mice

**Figure 4:**
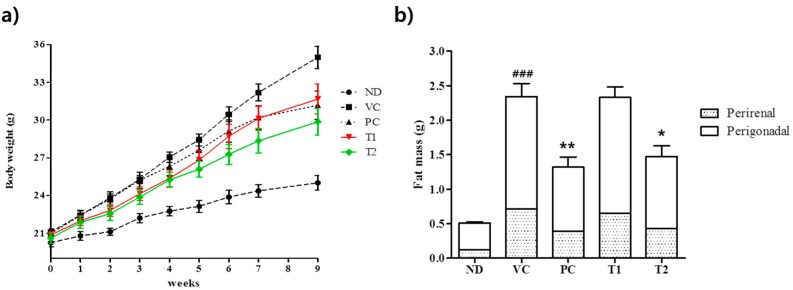
Effects of *A. melanocarpa* extract on body weights (**a**). Perirenal and perigonal fat masses (**b**) of mice fed a HFD for eight weeks. Results are presented as means ± SDs (*n* = 12). Weights in the *A. melanocarpa*-treated and Orlistat-treated HFD groups were significantly lower than those in the HFD group; ^###^ *p* < 0.001 vs. the ND group; * *p* < 0.05 and ** *p* < 0.01 vs. the VC group. Group I (ND), normal diet group; Group II (VC), high-fat diet group; Group III (PC), Orlistat-treated group; and Group IV (T1), 100 mg/kg b.w. *A. melanocarpa* extract-treated group; Group V (T2), 200 mg/kg b.w. *A. melanocarpa* extract-treated group.

HFDs promote significant expansion of adipose tissue in animal models of diet-induced obesity (DIO) and are often used to study obesity [33]. In the present study, consumption of a HFD for eight weeks caused significant increases in body, perirenal, and perigonadal weights (Figure 4), and mean body weight gain by mice fed the HFD was greater than observed in the ND (normal diet) group (Figure 4A). In fact, a 1.4-fold increase in body weight gain was observed in the VC (HFD + vehicle) group as compared with the ND group. On experimental week 9, after *A. melanocarpa* extract had been administered for eight weeks, mean body weight and perirenal and perigonadal fat mass gains were significantly lower in *A. melanocarpa*-treated HFD mice than in HFD controls. Weight gain rate of T2 group was slower than those of VC group, and mean body weight of T2 was significantly less than those of VC at seven and eight weeks of treatment. As was observed in the Orlistat-treated HFD-fed group (PC group), at 100 mg/kg/day *A. melanocarpa* extract reduced mean body weight by 30% as compared with HFD controls (the VC group). Orlistat is a gastrointestinal lipid uptake inhibitor and has been shown to have a positive effect on HFD induced obesity in murine models [34]. Notably, in the present study, greater hypolipidemic effects (as regards weight gain and adiposity) were observed for 200 mg/kg of *A. melanocarpa* extract than 15 mg/kg Orlistat in our HFD-induced murine model. Perirenal and perigonadal fat masses in the VC group were 3.5- and 5.6-fold, respectively, those observed in ND mice (Figure 4b). Furthermore, *A. melanocarpa* extract administered at 100 or 200 mg/kg dose-dependently attenuated perirenal and perigonadal fat mass increases in HFD-fed mice. In particular, T2 (200 mg/kg body weight *A. melanocarpa*) treatment inhibited HFD-induced increases in perirenal and perigonadal adipose tissue weights by 60% and 50%, respectively. The amount of food consumption was not significantly different between the groups during the experiment periods (Table 1).

#### 3.3.2. Effect of *A. melanocarpa* Extract on Serum Lipid Levels in HFD Mice

**Figure 5:**
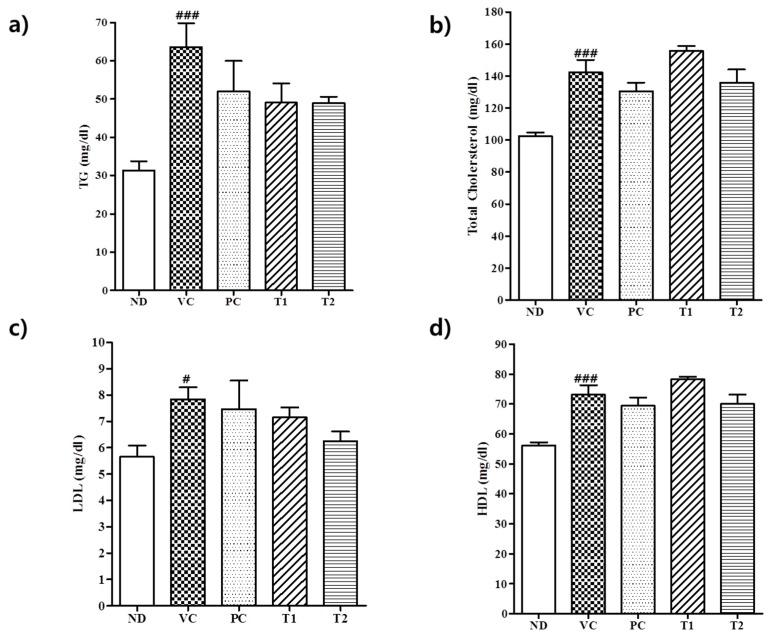
Effect of *A. melanocarpa* extract on serum lipid levels in HFD-induced obese mice. (**a**) Serum triglyceride (TG) levels. (**b**) Serum total cholesterol (TC) levels. (**c**) Serum low-density lipoprotein cholesterol (LDL) levels. (**d**) Serum high-density lipoprotein cholesterol (HDL) levels. Results are presented as means ± SDs (*n* = 12); ^#^ *p* < 0.05 and ^###^ *p* < 0.001 vs. the ND group. Group I (ND), normal diet group; Group II (VC), high-fat diet group; Group III (PC), Orlistat-treated group; and Group IV (T1), 100 mg/kg b.w. *A. melanocarpa* extract-treated group; Group V (T2), 200 mg/kg b.w. *A. melanocarpa* extract-treated group.

Feeding the HFD to male C57BL/6J mice rapidly increased TG, TC, HDL, and LDL serum levels (Figure 5). The serum concentrations of TG, TC, HDL, and LDL were 106%, 42%, 30%, and 37% higher in the VC group than in the ND group. HFD-induced obesity in mice has been shown to be associated with changes in lipid metabolism [35], and it has been reported TG serum levels are significantly higher in HFD mice and suggested increased TG synthesis is critical for the developments of obesity and hyperlipidemia [35,36]. Inhibiting TG synthesis is considered an effective strategy for obesity reduction because excessive TG accumulation results in obesity [37]. In the present study, serum concentrations of TG, TC, HDL, and LDL in HFD fed mice were remarkably reduced by T1 (100 mg/kg *A. melanocarpa*) by 45%, 23%, 23%, and 71% versus the VC group. However, no significant difference was observed between TC and HDL serum levels in the T1 (100 mg/kg *A. melanocarpa*)-treated and VC control groups. The TC/HDLC ratio has been reported to be a primary risk factor of dyslipidemia and coronary heart disease [38]. The value of this ratio in the high-fat diet group was 1.99, whereas in the T2 group it was only 1.07, which was actually much lower than observed in the VC group. These observations suggest *A. melanocarpa* extract might markedly inhibit the development of obesity and hyperlipidemia in diet-induced obese mice. Despite the significant increase in the level of serum TG and TC in the animals of VC group, the induction of liver steatosis was not found in all treatment groups (including VC). It seems that high fat chew alone was not sufficient to induce pathologic change in the liver (Supplement Data 1).

#### 3.3.3. Effects of *A. melanocarpa* Extract on HFD-Induced Glucose Intolerance and Insulin Resistance

**Figure 6:**
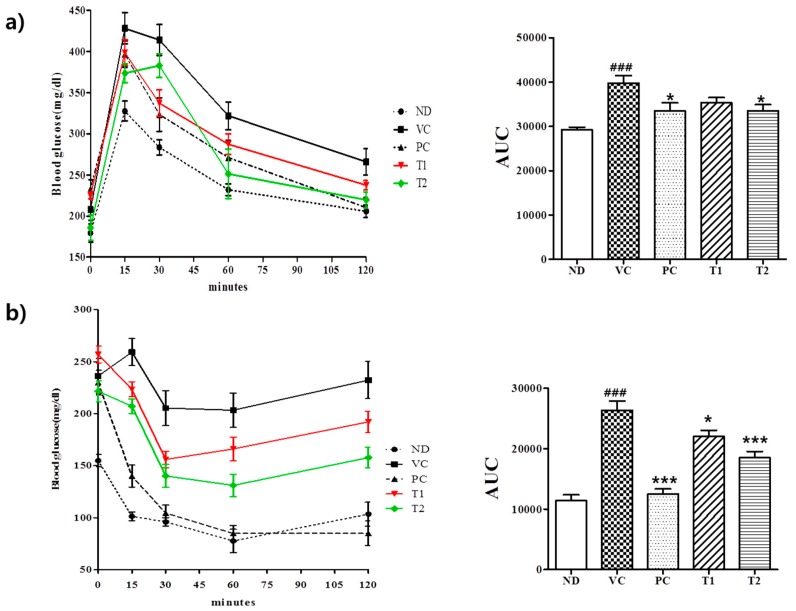
Effect of *A. melanocarpa* extract on oral glucose tolerance test (OGTT) and the respective AUC (**a**) and intraperitoneal insulin tolerance test (IPITT) and the respective AUC (**b**). Results are presented as means ± SDs (*n* = 12); ^###^ *p* < 0.001 vs. the ND group; * *p* < 0.05 and *** *p* < 0.001 vs. the VC group. Group I (ND), normal diet group; Group II (VC), high-fat diet group; Group III (PC), Orlistat-treated group; and Group IV (T1), 100 mg/kg b.w. *A. melanocarpa* extract-treated group; Group V (T2), 200 mg/kg b.w. *A. melanocarpa* extract-treated group.

Obesity is commonly associated with increased risks of developing insulin resistance and hyperinsulinemia [39]. Furthermore, it has been reported weight loss by obese patients improves glycemic control and reduces risk factors of type 2 diabetes [39,40]. However, the molecular basis responsible for the link between obesity and type 2 diabetes remains poorly understood. Adipose tissue is known to secrete various proteins, termed adipocytokines, and some of these are considered to be implicated in insulin sensitivity [41]. To determine whether *A. melanocarpa* extract ameliorates insulin resistance and regulates glucose metabolism, oral glucose and intraperitoneal insulin tolerance tests were performed on C57BL/6J mice. As shown in Figure 6, feeding a high-fat diet to mice resulted in severe glucose intolerance and insulin resistance, characterized by increases in areas under blood glucose curves and integrated AUCs. During OGTT, blood glucose in the VC group increased to 223 mg/dL after 15 min from a baseline value of 429 mg/dL (Figure 6a). The AUC value in the VC group was also 36% higher than that in the ND group. The T1 and T2 groups showed better glucose tolerance than the VC group as the AUCs of blood glucose curves were 43% and 57% lower, respectively. Furthermore, glucose levels in the VC group failed to return to baseline after 120 min, whereas in the T1 and T2 groups, blood glucose levels diminished more rapidly than in VC group as determined by IPITT (Figure 6b). In mice treated with 100 or 200 mg/kg *A. melanocarpa* administered insulin i.p., blood glucose levels decreased to 157 and 141 mg/dL, respectively, from baseline values of 262 mg/dL and 223 mg/dL at 30 min. AUC-OGTT and AUC-IPITT values in the PC group (positive control) mice were 58% and 86% less than those in the VC group, respectively. This observation suggests that the *A. melanocarpa* extract might reduce insulin resistance and improve insulin sensitivity.

## 4. Conclusions

The present work provides direct evidence of the beneficial effects of *A. melanocarpa* extract on diet-induced obesity in mice. Phenolic compounds isolated from *A. melanocarpa* extract, which included cyanidin glycosides and caffeic acid derivatives, were found to suppress adipogenesis by blocking major adipocyte marker proteins, such as, C/EBPα, PPARγ, and SREBP1c in 3T3-L1 preadipocytes. Furthermore, *A. melanocarpa* extract markedly inhibited the development of obesity and hyperlipidemia in HFD-induced obese mice, and *A. melanocarpa* extract treatment induced body weight changes were accompanied by improved glucose tolerance and insulin sensitivity in these obese mice. Our results suggest that *A. melanocarpa* may have positive health effects associated with the prevention and/or treatment of obesity.

## Figures and Tables

**Table 1:** The average food consumption per animal in each treatment group during pair feeding. The amount of food consumption was not significantly different between the groups.

Week	ND	VC	PC	T1	T2
	gram per animal				
1	2.35	2.20	2.10	2.10	2.20
2	2.00	2.25	2.55	2.15	2.20
3	1.95	2.30	2.20	2.20	2.20
4	2.40	2.40	2.40	2.25	2.05
5	2.95	2.50	2.90	2.30	2.15
6	2.45	2.70	2.40	2.60	2.45
7	2.65	2.50	2.15	2.25	2.45
8	2.55	2.65	2.35	2.10	2.30
Average	2.42	2.44	2.38	2.25	2.25
S.D.	0.33	0.18	0.26	0.16	0.14

Food consumption (g) = Full feeder-Empty feeder/day/number of mice per cage. ND, normal diet group; VC, high-fat diet group; PC, Orlistat-treated group; T1, 100 mg/kg b.w. A. melanocarpa extract-treated group; T2, 200 mg/kg b.w. A. melanocarpa extract-treated group.

## Supplementary Materials

The following are available online at http://www.mdpi.com/2072-6643/10/11/1734/s1.

## Abbreviations

PPARγ	peroxisome proliferator-activated receptor γ
C/EBPα	CCAAT/enhancer binding protein α
SREBP1c	Sterol regulatory element binding protein 1c
FAS	Fatty acid synthase
aP2	Adipocyte fatty-acid–binding protein
TG	Triglyceride
TC	Total cholesterol
LDLC	Low-density lipoprotein cholesterol
HDLC	High-density lipoprotein cholesterol
OGTT	Oral glucose tolerance test
IPITT	Intraperitoneal insulin tolerance test
