# Supplementary material for: Chokeberry Extract and Its Active Polyphenols Suppress Adipogenesis in 3T3-L1 Adipocytes and Modulates Fat Accumulation and Insulin Resistance in Diet-Induced Obese Mice
Source: Nutrients. 2018 Nov 12;10(11):1734. doi: 10.3390/nu10111734 (PMC6266992; doi:10.3390/nu10111734)

Chokeberry extract and its active polyphenols suppress adipogenesis in 3T3-L1 adipocytes and modulates fat accumulation and insulin resistance in diet-induced obese mice

Supplement data 1. Effect of *A. melanocarpa* extract on the histological changes of High Fat Diet -induced obese mice. Hepatocytes staining was carried out with the hematoxylin and eosin staining method. ND, normal diet group; VC, high-fat diet group; PC, Orlistat-treated group; T1, 100 mg/kg b.w. *A. melanocarpa* extract-treated group; T2, 200 mg/kg b.w. *A. melanocarpa* extract-treated group.

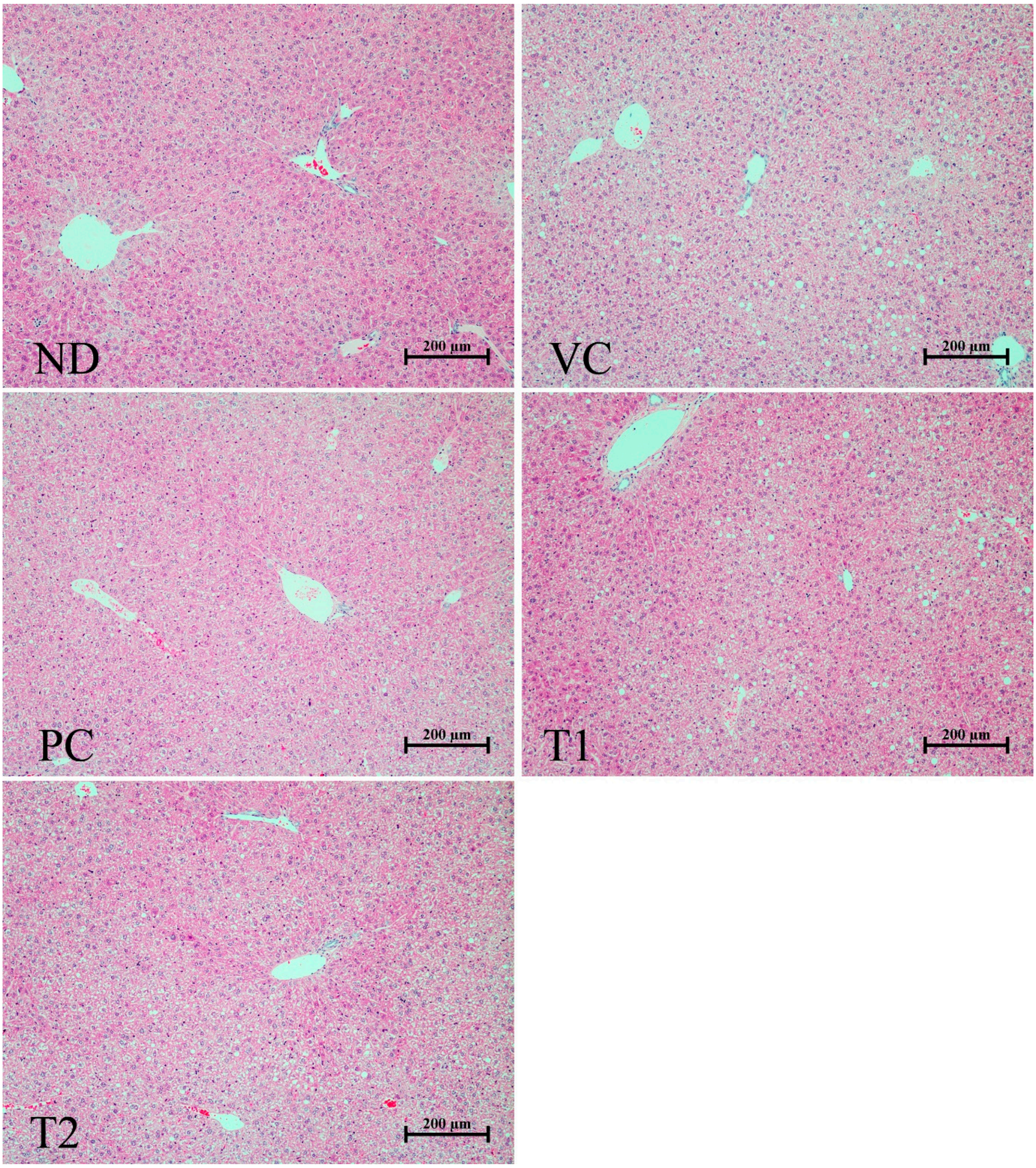

Supplement: Supplementary file 1 [file nutrients-10-01734-s001.pdf]
